# Antifungal Activity of Volatile Organic Compounds Produced by *Bacillus methylotrophicus* and *Bacillus thuringiensis* against Five Common Spoilage Fungi on Loquats

**DOI:** 10.3390/molecules25153360

**Published:** 2020-07-24

**Authors:** Chao-Nan He, Wan-Qiong Ye, Ying-Ying Zhu, Wen-Wen Zhou

**Affiliations:** College of Biosystems Engineering and Food Science, Zhejiang Key Laboratory for Agro-Food Processing, National-Local Joint Engineering Laboratory of Intelligent Food Technology and Equipment, Zhejiang University, Hangzhou 310058, China; hechaonan00@outlook.com (C.-N.H.); yeeewq@zju.edu.cn (W.-Q.Y.); 3160100469@zju.edu.cn (Y.-Y.Z.)

**Keywords:** *Bacillus methylotrophicus*, *Bacillus thuringiensis*, biocontrol, loquats, spoilage fungi, volatile organic compounds

## Abstract

Loquat fruit is one of the most perishable fruits in China, and has a very limited shelf life because of mechanical injury and microbial decay. Due to an increasing concern about human health and environmental security, antagonistic microorganisms have been a potential alternative for fungicides to control postharvest diseases. In this work, the antifungal effect of volatile organic compounds (VOCs) produced by *Bacillus methylotrophicus* BCN2 and *Bacillus thuringiensis* BCN10 against five postharvest pathogens isolated from loquat fruit, *Fusarium oxysporum*, *Botryosphaeria* sp., *Trichoderma atroviride*, *Colletotrichum gloeosporioides,* and *Penicillium expansum* were evaluated by in vitro and in vivo experiments. As a result, the VOCs released by BCN2 and BCN10 were able to suppress the mycelial growth of all targeted pathogens according to inhibition ratio in the double petri-dish assay as well as disease incidence and disease diameter on loquat fruits. The main volatile compounds were identified by solid-phase microextraction (SPME)-gas chromatography. These VOCs produced by the two strains played complementary roles in controlling these five molds and enabled loquat fruits to keep fresh for ten days, significantly. This research will provide a theoretic foundation and technical support for exploring the functional components of VOCs applicable in loquat fruit preservation.

## 1. Introduction

Loquat (*Eriobotrya japonica*) is widely cultivated in the subtropical regions of southern China, Japan, northern India, Israel, and the Mediterranean. Loquat fruits are highly perishable products, and generally can only keep fresh for 2 to 3 days at room temperature. Fruit provides an ideal substrate for the growth of pathogen microorganisms after harvest, such as the wound-invading fungi *Botrytis* spp., *Monilinia* spp. [1], *Penicillium expansum*, *Botrytis cinereal*, and *Colletotrichum acutatum* on apple [2], and *Penicillium digitatum* and *Penicillium italicum* on citrus [3]. Loquat fruit is susceptible to mechanical injury and microbial decay, which limits its storage period and marketing life.

Postharvest diseases cause considerable economic losses to harvested fruits and vegetables during transportation and storage. Synthetic fungicides are primarily used to control postharvest decay. However, the recent trend is shifting toward safer and more eco-friendly alternatives for the control of postharvest decay. Among various biological approaches, the application of antagonistic microorganisms is becoming more and more popular throughout the world. Several postharvest diseases can now be controlled by microbial antagonists [4].

Although some mechanisms by which microbial antagonists suppress the postharvest diseases are still unknown, competition for nutrients and space is the most widely accepted mode of action [5]. In addition, the production of antibiotics [6], direct parasitism, and possibly induced resistance [7] in the harvested commodity are also probable mechanisms. Microbial antagonists are applied either before or after harvest, but postharvest applications are more effective than preharvest applications [4].

The release of volatile organic compounds (VOCs) by some soil microbes has been reported to promote plant growth [8], display nematicidal activity [9], and induce systemic resistance in crops [10]. Headspace solid-phase microextraction (HS-SPME), a promising method for extraction of volatile compounds, is possible to identify characteristic VOCs for any bacterial species. The VOCs identified belong mainly to the alcohol group (ethanol, 3-methylbutan-1-ol, 2-methylbutan-1-ol, 2-phenylethanol), to the esters (ethyl acetate, ethyl octanoate) [11], and aldehydes (2-methylhex-2-enal, 2-isopropyl-5-methylhex-2-enal) [12]. *Bacillus* has been proven to produce nonvolatile or volatile substances which have inhibitory effects against *Fusarium oxysporum* [13,14], *Botryosphaeria berengeriana* [15], *Trichoderma* sp. [16], *Colletotrichum gloeosporioides* [17,18], and *Penicillium* sp. [19]. Volatile organic compounds responsible for the antifungal effect include 2,3,6-trimethylphenol, nonan-2-one, decan-2-one, dodecan-2-one, undecan-2-one, 2-methylpyrazine, etc. and effective protection against fungi is often not the action of one substance but a combination of many compounds. However, there are few broad-spectrum antifungal strains against postharvest diseases, not to mention relevant volatile data applied to loquat fruit.

The storage of loquat fruit is very difficult and there are few biological control methods. New *Bacillus* strains BCN2 and BCN10 were reported for the first time to show the excellent biocontrol effects on the spoilage of loquat fruits. The combined application of the two strains can provide the widest antifungal spectrum in loquat preservation. Volatile organic compounds produced by *Bacillus methylotrophicus* BCN2 and *Bacillus thuringiensis* BCN10 are key ingredients against postharvest pathogens, and the non-contact application of spore-forming bacteria also provides a guarantee for food safety and further commercial promotion. 

The objectives of this study were: (i) to evaluate the antifungal activity of VOCs produced by *Bacillus* BCN2 and BCN10 against five postharvest pathogens by in vitro and in vivo tests; (ii) to identify the compounds emitted by the two *Bacillus* strains with HS-SPME-gas chromatographic technique; (iii) to assess the inhibitory effect of VOCs produced by the two *Bacillus* species on natural decay of loquat fruit. This research will provide the theoretic foundation and technical support for exploring the functional components of VOCs applicable in loquat fruit preservation.

## 2. Results

### 2.1. Antagonistic Activities of VOCs Produced by BCN2 and BCN10 against Five Molds on Double Petri-Dishes

Data from the double petri-dish assays indicated that VOCs in the plate inoculated with both *Bacillus methylotrophicus* BCN2 and *Bacillus thuringiensis* BCN10 inhibited the mycelia growth of all five tested pathogens (*Fusarium oxysporum*, *Botryosphaeria* sp., *Penicillium expansum*, *Trichoderma atroviride*, *Colletotrichum gloeosporioides*), while the bacterial VOCs from either strain were unable to kill all these five pathogens (Table 1). BCN2 showed an inhibitory effect against *C. gloeosporioides, Botryosphaeria* sp., and *F. oxysporum.* Differently, BCN10 had an antifungal effect against *Botryosphaeria* sp., *P. expansum, T. atroviride,* and *C. gloeosporioides.*

For BCN2, the inhibition ratio against *C. gloeosporioides* was about 41% compared with the control, and those against *F. oxysporum* and *Botryosphaeria* sp. were from 10% to 20%, suggesting VOCs produced by BCN2 had a significant inhibitory effect on fungal mycelia. On the other hand, mycelial growth inhibition of BCN10 against *Botryosphaeria* sp. was 47%, *P. expansum* and *T. atroviride* were about 10% to 20%, respectively, and that against *C. gloeosporioides* was less than 10% but still above zero. Therefore, in terms of postharvest pathogens, BCN10 had a wider inhibitory spectrum than BCN2, and BCN2 was more targeted in the control of loquat anthracnose caused by *C. gloeosporioides* than BCN10.

### 2.2. Antagonistic Activities of VOCs Produced by BCN2 and BCN10 on Loquat Fruit

**Figure 1:**
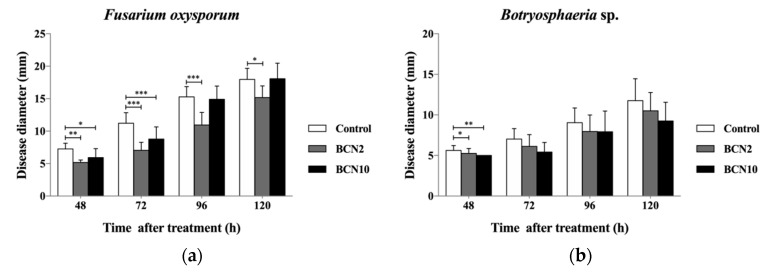

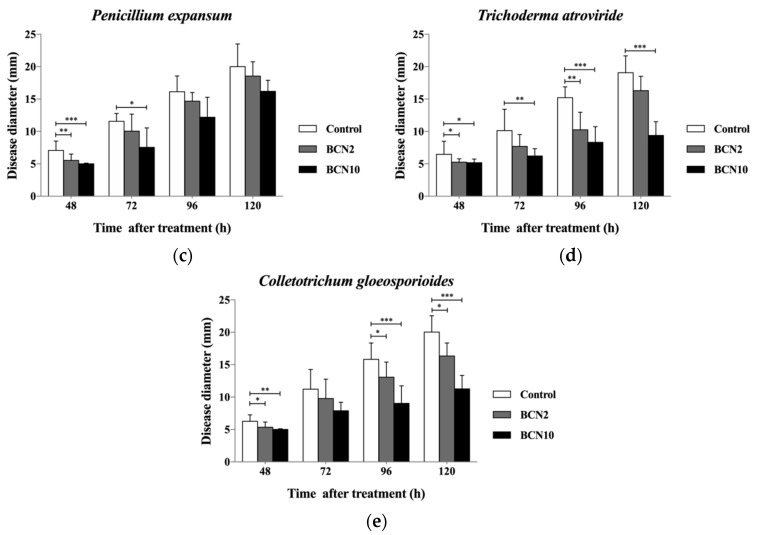
Disease diameter of loquat fruit with treatment of volatile organic compounds produced by two *Bacillus* strains. The loquat fruits were inoculated with *Fusarium oxysporum* (**a**), *Botryosphaeria* sp. (**b**), *Penicillium expansum* (**c**), *Trichoderma atroviride* (**d**), and *Colletotrichum gloeosporioides* (**e**) at 25 °C and 85% RH. * *p* <0.05, ** *p* <0.01, *** *p* <0.001 vs control, according to Tukey’s HSD test.

**Figure 2:**
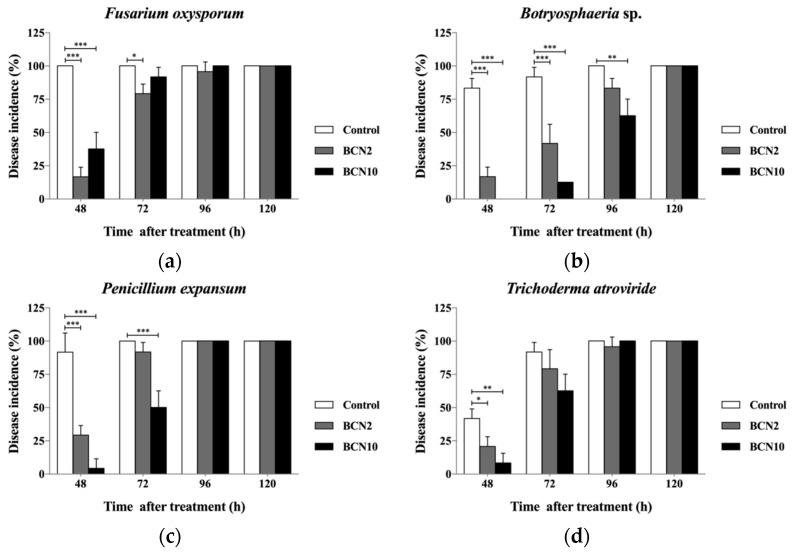

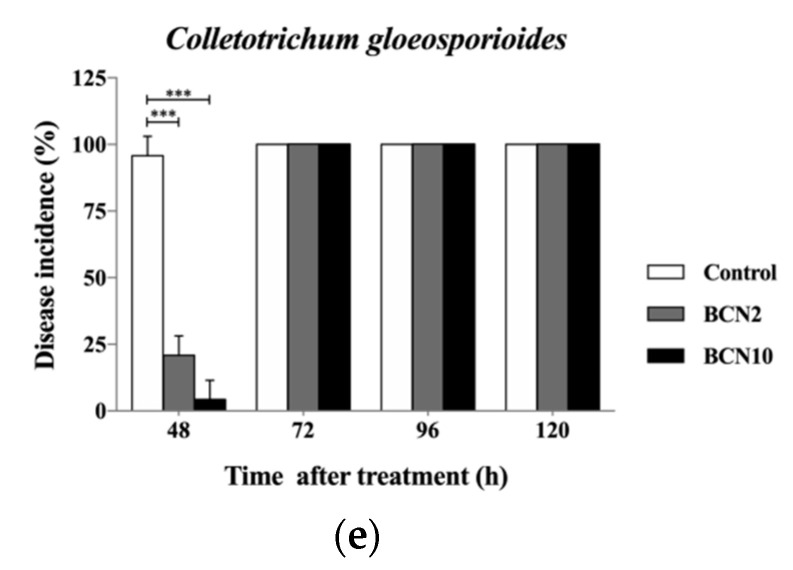
Disease incidence (percent of rotten fruits) of loquat fruit with the treatment of volatile organic compounds produced by two *Bacillus* strains. The loquats were artificially inoculated with *Fusarium oxysporum* (**a**), *Botryosphaeria* sp. (**b**), *Penicillium expansum* (**c**), *Trichoderma atroviride* (**d**), and *Colletotrichum gloeosporioides* (**e**) at 25 °C and 85% RH. * *p* <0.05, ** *p* <0.01, *** *p* <0.001 vs control, according to Tukey’s HSD test.

The VOCs produced by *B. methylotrophicus* BCN2 and *B. thuringiensis* BCN10 had an inhibitory effect against all tested pathogens (Figure 1 and Figure 2). VOCs produced by both strains were able to suppress the mycelial growth of all molds significantly according to Tukey’s HSD test and effectively lower disease incidence of all tested fungi in the first three days. Except for *F. oxysporum,* BCN10 performed better than BCN2 in controlling mycelial growth of the tested pathogens. Even until 5 days later, the reduced disease diameters of *T. atroviride* and *C. gloeosporioides* with BCN10 treatment were extremely significant. On the other hand, BCN2 significantly inhibited the growth of *F. oxysporum* all through the first 5 days after inoculation. Consistent with the result of disease diameter, BCN10 treatment led to lower disease incidence of the same four tested pathogens than BCN2. Therefore, the VOCs generated by BCN10 were possibly more antagonistic to some extent. The antagonistic effect of VOCs produced by BCN2 and BCN10 against five molds on loquat fruit was slightly stronger than that on double dishes because the volatile substances may not only have a direct antimicrobial effect on loquat but also play a role in inducing systemic acquired resistance to confer the long-lasting protection on fruit.

### 2.3. Gas Component Analysis of VOCs from BCN2 and BCN10

The headspace analysis indicated that *B. methylotrophicus* BCN2 and *B. thuringiensis* BCN10 could create diverse volatile profiles including alcohols, phenols, ketones, hydrocarbons, aldehydes, esters, acids, pyrazines, and other organic compounds (Table 2). Twenty-nine compounds were detected in the VOCs produced by BCN2, and in the meantime, thirty compounds were found in the VOCs of BCN10 compared to the control.

### 2.4. Decay Control of VOCs Produced by BCN2 and BCN10 in Natural Postharvest Storage of Loquat Fruits

As shown in Table 3, the VOCs generated by both *B. methylotrophicus* BCN2 and *B. thuringiensis* BCN10 had a strong inhibitory effect on the natural decay of loquat fruit. The treated loquats began to rot on the fifth day while the first rotten loquat found in control groups appeared on the second day, which means the VOCs from the two *Bacillus* strains enabled the loquat to keep fresh for more than 3 days at 25 °C and 85% RH. And on the tenth day, the disease incidence of the combined *Bacillus* group was 20.19%, which would still be low, compared to 54.17% of the control group. Overall, VOCs produced by BCN2 and BCN10 had an obvious inhibitory effect, while combined strains performed slightly better than BCN2 or BCN10 individually. Compared to the single bacteria treatment, the disease spots on the fruit were smaller, and the fruit was harder in the treatment group of the combined strains.

Until now, there have been few good methods available to preserve loquat fruit. Several plant essential oil components have been reported to be a potential natural preservative for fruits or other foodstuffs [20]. A previous study [21] showed that essential oil components like citral and carvacrol at 250 ppm could exhibit a fungistatic activity and inhibit mycelium growth. However, in our attempt to use essential oil as a positive control, some turned out to be not effective enough in controlling postharvest pathogens from loquat fruit.

## 3. Discussion

The biocontrol efficiency of BCN2 and BCN10 on loquat fruit was better than in the double petri-dish experiment. We speculated that the treatment enhanced activities of defense-related enzymes including chitinase, β-1,3-glucanase, phenylalanine ammonialyase, peroxidase, and polyphenoloxidase, and promoted accumulation of H_2_O_2_. With the release of volatile compounds of bacteria, total phenolic content and 2,2-diphenyl-1-picrylhydrazyl radical scavenging activity in loquat fruit might also increase, which changed the antioxidative activity and reduced the disease incidence in fruit. In this study, since *Bacillus* strains did not directly contact with loquat fruit, that means VOCs produced were responsible for the biocontrol efficiency. We did test some enzymes in loquat fruits after bacterial VOC’s treatment, though the data were instable, the trends were consistent with our speculations. Previous work has already reported that *Bacillus* spp. could enhance the activity of defense enzymes in rice [22], tomato [23], and peach [24], etc. There are a few reports showing that bacterial VOC can influence the activity of some defense enzymes of fruit and antioxidant enzymes of plant pathogens significantly [25,26,27]. Although the mechanisms by which microbial antagonists control postharvest diseases have not been clearly elucidated, induced disease resistance has been inferred to be one of the major modes of their actions [4]. Chitinase hydrolyzes the β-1-4-linkage in chitin, which is an essential cell wall component of fungi, while β-1,3-glucanase directly degrades cell walls of pathogens or indirectly releases oligosaccharide and elicits defense reactions; therefore both enzymes are thought to be involved in plant defense mechanisms against fungal infection [28]. PAL is the first enzyme in the phenylpropanoid pathway leading to the biosynthesis of phenolics, phytoalexins, lignins and many other compounds associated with localized disease resistance in plants [29], while PPO and POD are both involved in lignification of host plant cells and considered as key enzymes related to defense reaction against pathogen infections [30]. The induction of these defense-related enzymes by different biocontrol agents has been observed in harvested apple, Chinese bayberry, loquat, and peach fruit, and is correlated to increased disease resistance and reduced disease severity [31,32,33,34].

In order to understand the nature of VOC produced by BCN2 and BCN10, SPME and GC-MS were used in couple. This simple and rapid technology for sampling volatile compounds at low concentrations in headspace analysis has been successfully used to characterize VOCs. VOCs are chemicals with low molecular weights (<300 Da), high vapor pressure and low water solubility, and terpenes, alkanes, and aromatic hydrocarbons are mostly identified by GC-MS analysis. These compounds include myrcene, limonene, geraniol, undecane, cyclohexane, ethylbenzene, toluene, and so forth, and many antifungal volatiles come from plant essential oils [35,36,37]. The headspace analysis showed that 29 and 30 organic compounds were detected from BCN2 and BCN10, respectively, but not in the control. Although most of the compounds detected have already been reported in different *Bacillus* strains [38,39], methodologies applied to collect and detect VOCs should be taken into account, because it can also influence the results and often confuse the comparison between different studies. Moreover, these data not only depend on compound concentration but also on the fiber affinity and the detector sensing to the different analytes.

As proposed by Maruzzella [40], the activities of antifungal VOCs decrease in the following order: organic acids > aldehydes > alcohols > ethers > ketones > esters > lactones. However, in other works, when a single molecule was tested on molds, this order was not absolute. Antifungal activity of VOCs has been reported to be associated with the functional group [41], and it is dependent on the hydrophobicity of the solute, which affects the depth of penetration into the bilayer, and the induced changes in the physic-chemical properties [40]. It is well known that the effect of a lipophilic compound on the integrity of a membrane depends on its location in the membrane, where it has been accumulated, resulting in lower membrane integrity and an increase in the proton passive flux-across the membrane. This could be the case of carbon disulfide that dramatically suppressed the growth of pathogens in loquat fruit.

Among these VOCs, fatty acids with short chains are highly active compounds against fungi, probably due to their higher solubility in water [42]. In this study, acetic acid (a food additive with broad-spectrum antibiosis activity) and 2-methylpropanoic acid (a common antibacterial component in many plant essential oils) in *Bacillus* VOCs probably exhibit obvious effective antifungal activity. In contrast to fatty acids, alcohols are not selectively adsorbed and are mainly accumulated in the cell membrane, determining membrane functions inhibition, which in turn, is responsible for their antimicrobial activity [43]. In particular, 3-methylbutan-1-ol may be the effective compound in bacterial VOCs against fungal growth. Because some alcohols, such as 3-methylbutan-1-ol (also known as isoamyl alcohol), can be adsorbed on the spores’ surface and therefore adhere to it for a long time, resulting in spore germination inhibition [44].

Some articles reported antifungal activity analyses of selected molecules produced by single *Bacillus* species [14,38,45,46], and the activity of ketones was considered to be negatively correlated with the number of the carbon atoms, and thus nonan-2-one and decan-2-one, in particular, showed strong inhibition activity [14].

Other volatile molecules produced by *Bacillus* species have been tested as single compounds towards the fungal growth of other species, and the results showed significant antifungal activity. For example, aldehydes like o-anysaldehyde, nonanal, and n-decanal, could reportedly inhibit germ tube formation of *Alternaria alternate* [17], or mycelia growth of *B. cinerea* and *S. sclerotiorum* [45,46]. Furthermore, alcohols like hexan-1-ol, 2-ethylcyclohexanol, benzothiazole and dimethyl trisulphide exerted high fungicidal activity against *S. sclerotiorum* [46]. In addition, 2-methylpirazyne and β-benzeneethanamine showed antifungal effect against *C. gloesporoides* [15].

Inhibitory effects of VOCs produced by the two *Bacillus* strains *B. methylotrophicus* BCN2 and *B. thuringiensis* BCN10 on natural decay of loquat fruits were strongly manifested. The difference of disease incidence between experimental groups and control groups demonstrated that the VOCs emitted by both BCN2 and BCN10 could put off the decay of loquat till the fifth day, which was three days later than the control groups and enabled more loquat to keep fresh for ten days significantly. Future research will be directed toward designing agriculturally practical ways to efficiently use antifungal volatiles to reduce fruit decay under storage conditions.

The two *Bacillus* strains are complementary in the role of antagonism against the pathogens. Mixed cultures of the microbial antagonists appear to provide better control of postharvest diseases over individual cultures. At the international level, different microbial antagonists such as *Debaryomyces hansenii*, *Cryptococcus laurentii*, *B. subtilis*, and *T. harzianum* are being used around the world. Biocontrol products such as Aspire, BioSave, Shemer, etc., have also been developed and registered. However, the biocontrol products available do not have much effect on loquat fruit. In our work, mixed cultures of the two *Bacillus* strains can reduce disease incidence by more than 30% in ten days, which is hard for existing biocontrol agents to achieve. Although the results of this technology are encouraging, we need to further explore potential applications on the commercial scale in different corners of the world [4].

## 4. Materials and Methods

### 4.1. Mircoorganisms Culture Media

The antagonistic strain BCN2 was identified as *B. methylotrophicus* (CGMCC NO. 16443) and BCN10 was identified as *B. thuringiensis* (CGMCC NO. 16675) by 16S rRNA sequencing (see 5. Patents). Stock cultures were stored at 4 °C and subcultured on Luria-Bertani culture at 374 °C for 12 h when required. The cells were collected in potassium phosphate buffer and adjusted to a final concentration of 10^8^ CFU ml/mL.

The target fungal strains *F. oxysporum*, *Botryosphaeria* sp., *T. atroviride*, *C. gloeosporioides,* and *P. expansum* were maintained on potato dextrose agar (PDA agar) plates at 25 °C for 3 days.

### 4.2. In Vitro Antagonistic Activities of VOCs from Two Bacillus Strains

The efficacy of the VOCs Produced by *B. methylotrophicus* BCN2 and *B. thuringiensis* BCN10 on the mycelium was tested by the double petri-dish assay. One compartment of the divided plates containing LB medium was inoculated with BCN2 and/or BCN10, except for control plates. Another compartment containing PDA medium was used for one type of these five fungal strains. The plates were inoculated at 25 °C for 3 days, and then the diameters of the fungal growth in different plates were measured. The inhibition percentage of mycelial growth was calculated on the basis of the difference between treatment and control according to the formula: (C – T/C) × 100, where C is the control value, and T is the measurement of the fungus in each antagonist-fungal set-up. The sample unit was represented by three replicates of each pathogen and antagonist interaction under each of the conditions mentioned.

### 4.3. In Vivo Antagonistic Activities of VOCs

One trial was conducted with loquat fruits to evaluate the antagonistic activities of VOCs produced by *B. methylotrophicus* BCN2 and *B. thuringiensis* BCN10. Loquat fruits, obtained in Mengzi County in Yunnan province, were selected without visible injuries and rots and homogeneous in maturity and size. Then loquats were disinfected by 0.2% sodium hypochlorite for 1–2 min and rinsed with deionized water. Each fruit was punched with a hole (5 mm in diameter and 3 mm in depth) on its surface using a sterilized perforator and inoculated with 20 µL suspension of 10^4^ CFU/mL fungal strains respectively, except for the control. 40 mL of LB inoculated with BCN2 or/and BCN10 was sealed in Ziploc bags at 37 °C for 12 h. Then the loquats inoculated with fungi were put inside the bags in piles of eight without direct contact with *Bacillus* culture vial. The control consisted of inoculated fruit placed in bags with no *Bacillus* culture vial. All the experiments were performed in triplicate for each pathogen and antagonist interaction in every condition mentioned (24 loquat fruits for each data). The percentage of rotten fruits (disease incidence) and the diameters of the disease speckles were determined after two days of storage and every day following at 25 °C and 85% RH.

### 4.4. HS-SPME GC-MS Analysis of VOCs

A qualitative and quantitative evaluation of VOCs produced by *B. methylotrophicus* BCN2 and *B. thuringiensis* BCN10 composition was done using HeadSpace solid phase microextraction (HS-SPME) coupled with gas chromatography-tandem mass spectrometry analysis (GC-MS) according to the method previously described by Di Francesco et al. (2015) with modifications. SPME fiber (2 cm—50/30 mm DVB/ CAR/PDMS, Supelco Inc, Bellefonte, PA, USA) was preconditioned according to manufacturer’s recommendations. The strain BCN2 or BCN10 was inoculated into 40 mL of LB medium in a 100 mL vial for 12 h at 37 °C and the vials were put inside the Ziploc bags with the fresh fruit, where the placement was the same as described in Section 4.3. The LB medium without inoculation was the control. The SPME fiber was inserted into the vial, fixed at about 1 cm above the surface of the fermentation liquid, and exposed to headspace in 50 °C hot water for 30 min to extract the VOCs. Trapped compounds were then thermally desorbed from the fiber for 2 min in the GC injection port at 250 °C in the split-less injection mode. For peak separation and detection, a Bruker GC 451 gas chromatograph equipped with a HP-5 fused silica capillary column (30 m by 0.25 mm inside diameter; 0.25 mm film thickness, J&W Scientific Inc., Folsom, CA, USA). The transfer line was heated at 250 °C, the ion source at 220 °C and carrier gas (He) flow rate was 1 mL/min. The mass spectrometer was operated in electron impact mode at 70 eV, scanning the range of 35/500 m/z in a full scan acquisition mode. The GC oven temperature was set at 40 °C for 4 min and then programmed to rise from 40 to 90 °C at 10 °C/min, from 90 to 160 °C at 5 °C/min and from 160 to 280 °C at 40 °C min [47]. GC-MS took cyclohexanone as the internal standard substance and made a quantitative test.

The identification of VOCs was confirmed by retention time and mass spectra comparison with identical standard compounds. The mass spectra of VOCs were compared with those in the NIST/EPA/NIH Mass Spectrometry Library with respect to the spectra in the NIST 11 MS databases. Blank sample analysis was performed under the same conditions in order to exclude interfering substances. All measurements were made with three replicates.

### 4.5. Effectiveness of VOCs Produced by BCN2 and BCN10 in Natural Storage of Loquats

Loquat fruits were selected without visible injuries and rots and homogeneous in maturity and size and simply cleaned by deionized water to remove surface blots without being disinfected. Plates inoculated with BCN2 or BCN10 were sealed in piles of ten in Ziploc bags at 37 °C for 12 h. Then the selected loquats were put inside the bags in piles of eight without direct contact with the bacteria culture vial, except for the control. The control consisted of eight fruits placed in bags and LB medium with no antagonists. The sample unit was represented by three replicates. The percentage of rotten fruits (disease incidence) was measured after three days at 25 °C and 85% RH.

### 4.6. Data Analysis

Data on the percentage of mycelial growth inhibition was calculated according to the formula described in Section 4.2. Disease incidence and the diameters of the disease speckles were analyzed at least in duplicate. Differences in the diameters of the disease speckles were evaluated using analysis of variance (ANOVA) with the JMP^®^8 statistical software (SAS Institute, Cary, NC, USA). Statistical significance was judged at the level *p* <0.05. When the analysis was statistically significant, the Tukey’s HSD Test was used for the separation of the means [48].

## 5. Patents

Zhou, W.-W.; He, C.-N.; Sun, Y.-F. A *Bacillus thuringiensis* strain for biological control and its application. Chinese Patent 2018, CN201811495512.4.

Zhou, W.-W.; He, C.-N.; Sun, Y.-F. The application of a *Bacillus methylotrophicus* strain with biocontrol activity. Chinese Patent 2018, CN201811217239.9.

## Figures and Tables

**Table 1:** Inhibition of volatile organic compounds from *Bacillus methylotrophicus* BCN2 and *Bacillus thuringiensis* BCN10 to mycelial growth of *Fusarium oxysporum*, *Botryosphaeria* sp., *Trichoderma atroviride*, *Colletotrichum gloeosporioides*, and *Penicillium expansum* at 28 °C for 3 days.

Pathogens	*Fusarium oxysporum*	*Botryosphaeria* sp.	*Penicillium expansum*	*Trichoderma atroviride*	*Colletotrichum gloeosporioides*
*Bacillus*					
BCN2	++	++	---	---	+++
BCN10	---	+++	++	++	+
BCN2+BCN10	++	+++	++	++	+++

--- means no inhibition effect; + means the inhibition effect is weak, and the inhibition rate of colony radius is less than 10%, affecting the growth of fungal hypha; ++ means the inhibition effect is obvious, and the inhibition rate of colony radius is between 10–20%; the inhibition effect of +++ is significant, and the inhibition rate of colony radius is more than 20%; the average is taken for three repetitions.

**Table 2:** Volatile compounds detected in the headspace from the *Bacillus* cultures inoculated in LB culture vial at 37 °C for 12 h.

	*Bacillus methylotrophicus* BCN2			*Bacillus thuringiensis* BCN10		
	RI ^a^		Conc ^b^	RI ^a^		Conc ^b^
Alcohols	434	Butan-1-ol	0.17 ± 0.02	430	Butan-1-ol	0.52 ± 0.04
	506	3-Methylbutan-1-ol	0.27 ± 0.10	513	3-Methylbutan-1-ol	0.56 ± 0.08
	671	Hexan-2-ol	0.45 ± 0.06	750	Phenylmethanol	0.05 ± 0.00
	864	Phenylethanol	2.05 ± 0.12	862	Phenylethanol	1.00 ± 0.08
Phenols	927	2,3,6-Trimethylphenol	0.37 ± 0.05			
	1256	Benzothiazole-5,7-ol	0.19 ± 0.11			
Ketones	412	Butan-2-one	0.07 ± 0.03	412	Butan-2-one	0.25 ± 0.05
	465	3-Hydroxybutan-2-one	1.43 ± 0.07	459	3-Hydroxybutan-2-one	1.80 ± 0.07
	918	Nonan-2-one	0.71 ± 0.12	403	Butane-2,3-dione	3.13 ± 1.08
	1076	Decan-2-one	0.41 ± 0.05	859	Acetophenone	0.95 ± 0.03
	1198	Undecan-2-one	0.22 ± 0.11	870	6-Methylheptan-2-one	0.06 ± 0.01
	1280	Dodecan-2-one	2.06 ± 0.98			
	1333	Tridecan-2-one	0.23 ± 0.08			
	1598	Pentadecan-2-one	0.19 ± 0.11			
Hydrocarbons	419	2-Methylpropane	0.39 ± 0.07	417	2-Methylpropane	3.13 ± 0.08
	530	Penta-1,3-diene	0.44 ± 0.06	533	Penta-1,3-diene	1.17 ± 0.00
	751	Toluene	1.21 ± 0.54	757	2,4-Diaminotoluene	0.45 ± 0.19
	840	Ethylbenzene	0.98 ± 0.23	868	Oct-1-ene	0.70 ± 0.04
	941	Propylbenzene	1.48 ± 0.08	1113	2-Methylnaphthalene	3.05 ± 0.51
	923	Isopropylbenzene	2.11 ± 0.53	1265	Dodecane	0.79 ± 0.07
	1407	Tetradecane	0.15 ± 0.06	1412	Tetradecane	0.34 ± 0.04
	1541	Pentadecane	5.98 ± 0.30	1971	Eicosane	0.16 ± 0.03
Aldehydes				811	Benzaldehyde	4.01 ± 0.17
Esters	423	Ethyl acetate	0.78 ± 0.19			
Acids	249	Acetic acid	1.54 ± 0.01	241	Acetic acid	0.75 ± 0.42
	477	2-Methylpropanoic acid	0.06 ± 0.02			
Other compounds	221	Carbon disulphide	0.34 ± 0.09	230	Carbon disulphide	0.17 ± 0.01
	614	2,6-Dimethylpyrazine	0.06 ± 0.01	745	Benzothiazole	0.29 ± 0.04
	855	2-Ethyl-3,5-dimethylpyrazine	0.67 ± 0.15	762	4-Methyl-1,3-benzenediamine	1.36 ± 0.03
	873	Tetramethylpyrazine	2.02 ± 0.07	881	2-(Methylthio)benzothiazole	0.45 ± 0.07
				807	2-Methoxyphenyl oxime	0.23 ± 0.18
				845	3-Ethyl-2,5-dimethylpyrazine	0.89 ± 0.01
				976	2-Pentylfuran	3.29 ± 0.16
				957	2,3,5-Trimethyl-6-ethylpyrazine	0.09 ± 0.04
				965	2,3-Diethyl-5-methylpyrazine	0.77 ± 0.43
				1132	2,5-Dimethyl-3-(3-methylbutyl)pyrazine	0.24 ± 0.05
				1147	2,3-Dimethyl-5-isopentylpyrazine	0.51 ± 0.14

^a^ RI, Retention indices; ^b^ Conc, concentration was expressed in microlitre per litre of fermentation broth, cyclohexanone was used as internal standard, and data listed were the mean of three assays ± SD.

**Table 3:** Inhibitory effect of volatile organic compounds produced by two *Bacillus* strains in the natural decay of loquat fruit.

Disease Incidence	3 d	5 d	10 d	15 d
Control	12.50%	20.83%	54.17%	100.00%
BCN2	0.00%	4.17%	20.83%	87.50%
BCN10	0.00%	5.77%	22.34%	89.08%
BCN2+BCN10	0.00%	3.56%	20.19%	86.95%
